# The regulation of hydroxysteroid 17β-dehydrogenase type 1 and 2 gene expression in breast cancer cell lines by estradiol, dihydrotestosterone, microRNAs, and genes related to breast cancer

**DOI:** 10.18632/oncotarget.19136

**Published:** 2017-07-10

**Authors:** Erik Hilborn, Olle Stål, Andrey Alexeyenko, Agneta Jansson

**Affiliations:** ^1^ Department of Clinical and Experimental Medicine and Department of Oncology, Faculty of Health Sciences, Linköping University, Linköping, Sweden; ^2^ Department of Microbiology, Tumor and Cell Biology (MTC), Karolinska Institutet, Stockholm, Sweden; ^3^ National Bioinformatics Infrastructure Sweden, Science for Life Laboratory, Solna, Sweden

**Keywords:** breast cancer, HSD17B1, HSD17B2, miRNA

## Abstract

Aim. To investigate the influence of estrogen, androgen, microRNAs, and genes implicated in breast cancer on the expression of HSD17B1 and HSD17B2. Materials. Breast cancer cell lines ZR-75-1, MCF7, T47D, SK-BR-3, and the immortalized epithelial cell line MCF10A were used. Cells were treated either with estradiol or dihydrotestosterone for 6, 24, 48 hours, or 7 days or treated with miRNAs or siRNAs predicted to influence HSD17B expression Results and discussion. Estradiol treatment decreased *HSD17B1* expression and had a time-dependent effect on *HSD17B2 expression*. This effect was lost in estrogen receptor-α down-regulated or negative cell lines. Dihydrotestosterone treatment increased *HSD17B2* expression, with limited effect on *HSD17B1* expression. No effect was seen in cells without AR or in combination with the AR inhibitor hydroxyflutamide. The *miRNA-17* up-regulated *HSD17B1*, while *miRNA-210* and *miRNA-7-5p* had up- and down-regulatory effect and *miRNA-1304-3p* reduced *HSD17B1* expression. The *miRNA-204-5p, 498, 205-3p* and *579-3p* reduced *HSD17B2* expression. Downregulation of CX3CL1, EPHB6, and TP63 increased *HSD17B1* and *HSD17B2 expression*, while *GREB1* downregulation suppressed *HSD17B1* and promoted *HSD17B2* expression. Conclusion. We show that *HSD17B1* and *HSD17B2* are controlled by estradiol, dihydrotestosterone, and miRNAs, as well as modulated by several breast cancer-related genes, which could have future clinical applications.

## INTRODUCTION

Breast cancer is the malignant growth of cells in the breast tissue, and 10% of women will be diagnosed with breast cancer. Estrogen and androgen signaling are involved in regulating tumor growth and progression. Seventy to 80% of all breast cancers express the estrogen receptor (ER)α [1, 2] and 60-80% express the androgen receptor (AR) [3, 4]. Estrogen signaling by ERα results in cell proliferation and survival [5]. Androgens signaling through AR is primarily antiproliferative in ERα-positive tissues and AR is associated with improved outcome in ERα-positive breast cancer [6-10]. In postmenopausal women, the primary source of estrogen in the breast tissue is the product of local conversion mediated by aromatase, steroid sulfatase (STS) and hydroxysteroid 17β-dehydrogenase (HSD17B) 1 and 2 [11-17].

The primary role of HSD17B1 is to mediate the reduction of estrone to estradiol, dehydroepiandrosterone (DHEA) to androstenediol, and dihydrotestosterone into 3β-diol and 3α-diol [18, 19]. HSD17B2 catalyzes the oxidation of estradiol to estrone, testosterone to androstenedione and androstenediol to DHEA [20]. HSD17B1 is associated with adverse outcome [21-24], while HSD17B2 has been proposed to protect the tissue from steroid overexposure and is associated with improved outcome [14, 21]. Not much is known about the control of the expression of HSD17B1 and HSD17B2 in breast cancer, apart from that they are correlated with ERα expression [19, 22, 24], and that dihydrotestosterone can induce HSD17B2 expression in an AR-dependent manner in the breast cancer cell line T-47D [25].

MicroRNAs (miR)s are ∼21 nucleotides short inhibitory RNAs, involved in almost every part of carcinogenesis. MiRs have been estimated to regulate the expression of as many as 30% of all genes [26]. HSD17B1 has been shown to be under the regulation of miR-210 and 518c in placental cells [27]. To date, no study has been published examining the role of miRNAs regarding HSD17B1 and HSD17B2 in breast cancer.

### Aim

We aimed to investigate if estrogen and androgen-mediated signaling can influence the expression of *HSD17B1* and *HSD17B2* in breast cancer cell lines. Furthermore, we aimed to identify microRNAs responsible for the regulation of *HSD17B1* and *HSD17B2* and to identify genes which control the expression of *HSD17B1* and *HSD17B2* in breast cancer cell lines.

## RESULTS

### Estradiol signaling controls HSD17B1 and HSD17B2 mRNA expression

**Figure 1:**
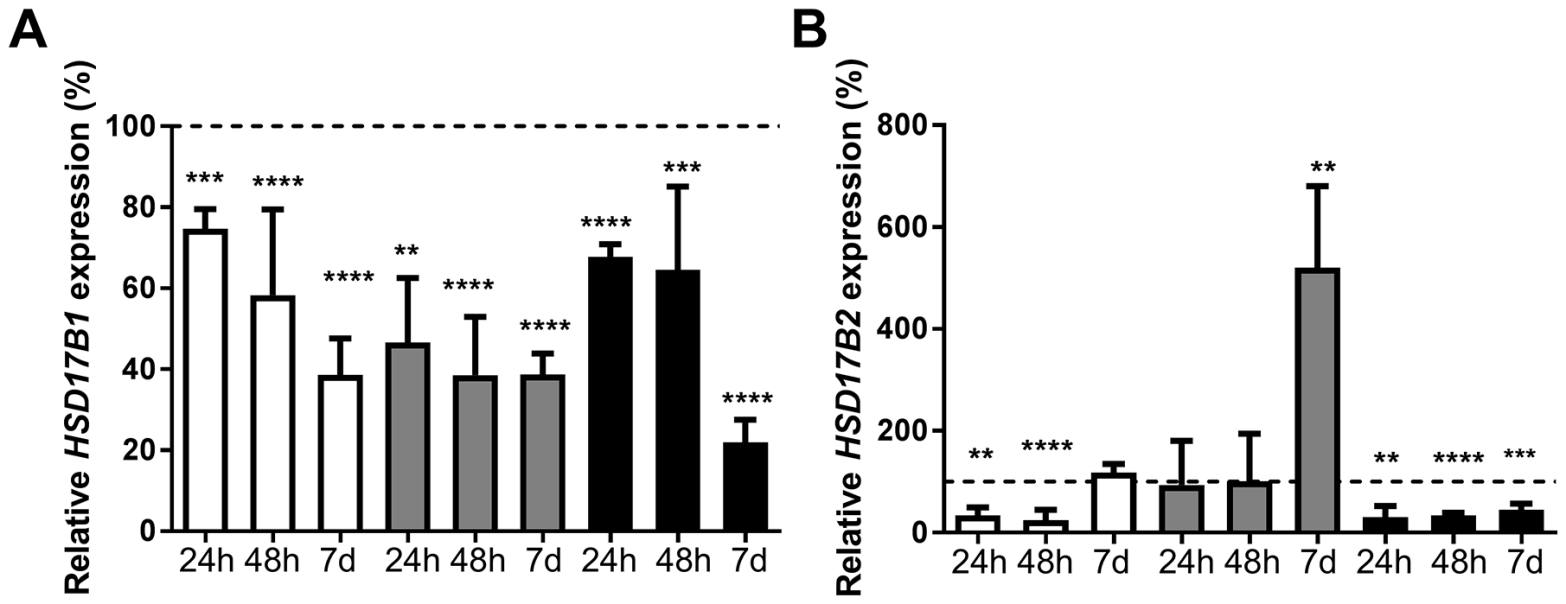
Relative HSD17B1 **(A)** and HSD17B2 **(B)** expression following estradiol treatment in ZR-75-1 (white), MCF7 (gray) and T-47D (black), for 24h n=3, 48h n=6, and 7 days n=5. All effects on the relative HSD17B1 and HSD17B2 expression are compared to ethanol-treated controls from the same time point and cell line as the treated sample. Error bars represent standard derivation.

ZR-75-1, MCF7, and T-47D were treated with 5nM and 10nM estradiol for 6 hours, 24 hours or 48 hours. Since the effect of 5nM was similar to 10nM (data not shown), 5nM was used when cells were treated for 7 days. The expression of *HSD17B1* and *HSD17B2* was compared with ethanol-treated cells for the corresponding time point. At 6 hours, there was no significant change regarding *HSD17B1* or *HSD17B2* expression (data not shown). Following treatment with 5nM estradiol, the expression of *HSD17B1* in ZR-75-1 cells decreased to 75% at 24 hours, 58% at 48 hours and 38% at 7 days. In MCF7 cells there was a decrease to 47% at 24 hours, and a decrease to 39% at 48 hours and 7 days. In T-47D there was a decrease to 68% at 24 hours, 64% at 48 hours and 22% at 7 days, Figure 1A. The expression of *HSD17B2* was also influenced by estradiol treatment; in ZR-75-1 there was a reduction to 33% at 24 hours and 25% at 48 hours. There was no effect after 7 days. In MCF7 there was no change at 24 hours or 48 hours, but an increase to 520% at 7 days. In T-47D there was a reduction to 30% at 24 hours, 34% at 48 hours and 45% at 7 days, Figure 1B. In summary, estradiol stimulation appears to mediate a reduction of *HSD17B1* expression in all cell lines. Further, estradiol stimulation appears to reduce *HSD17B2* expression in ZR-75-1 and T-47D at 24h and 48 hours, while at 7 days in MCF-7 expression is greatly increased, and in ZR-75-1 the *HSD17B2* expression reverts to baseline.

**Figure 2:**
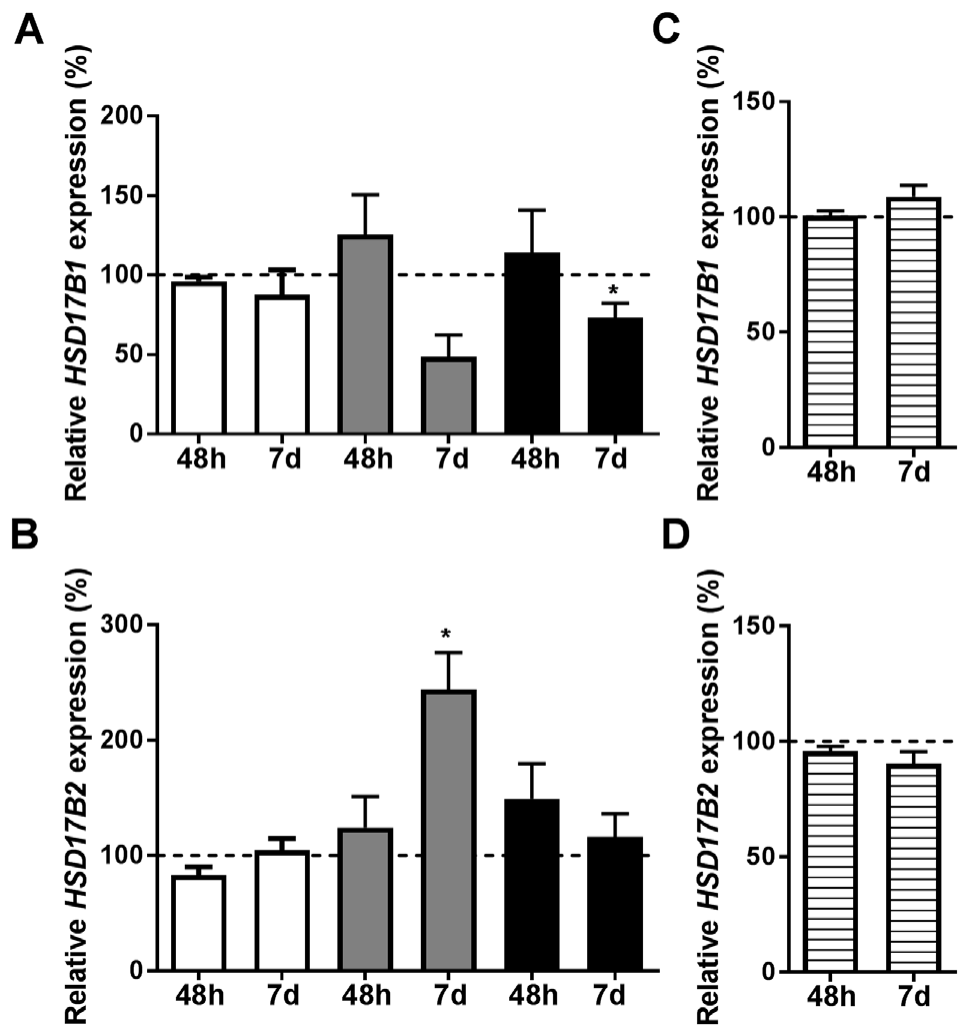
Relative HSD17B1 **(A, C)** and HSD17B2 **(B, D)** expression. (A, B) denotes the effects of estradiol treatment compared to ethanol treated control in ERα downregulated ZR-75-1 (white), MCF7 (gray), and T-47D (black), n=3 at 48 hours and 7 days, n=3. (C, D) denotes the effect of estradiol in SK-BR-3 (horizontal stripes), n=3. All effects on the relative HSD17B1 and HSD17B2 expression are compared to ethanol-treated controls from the same time point and cell line as the treated sample. Error bars represent standard derivation.

With the purpose of determining if the estradiol signaling was ERα-dependent, ERα was transiently downregulated. ERα mRNA levels remained suppressed at 7 days, Supplementary Figure 1. At 48 hours, there was no ERα protein expression in either cell line, Supplementary Figure 2. At 48 hours, neither *HSD17B1* nor *HSD17B2* was significantly altered by estradiol treatment, Figure 2. At 7 days, there was a significant reduction of *HSD17B1* in T-47D, Figure 2A, and there was an increase of HSD17B2 in MCF7, Figure 2B. Using the ERα-negative cell-line SK-BR-3 treated for 48 hours or 7 days with estradiol, we show no effect of estradiol on *HSD17B1* or *HSD17B2* expression, Figure 2C and 2D.

### Dihydrotestosterone signaling alters HSD17B2 expression in ERα-positive breast cancer cells

**Figure 3:**
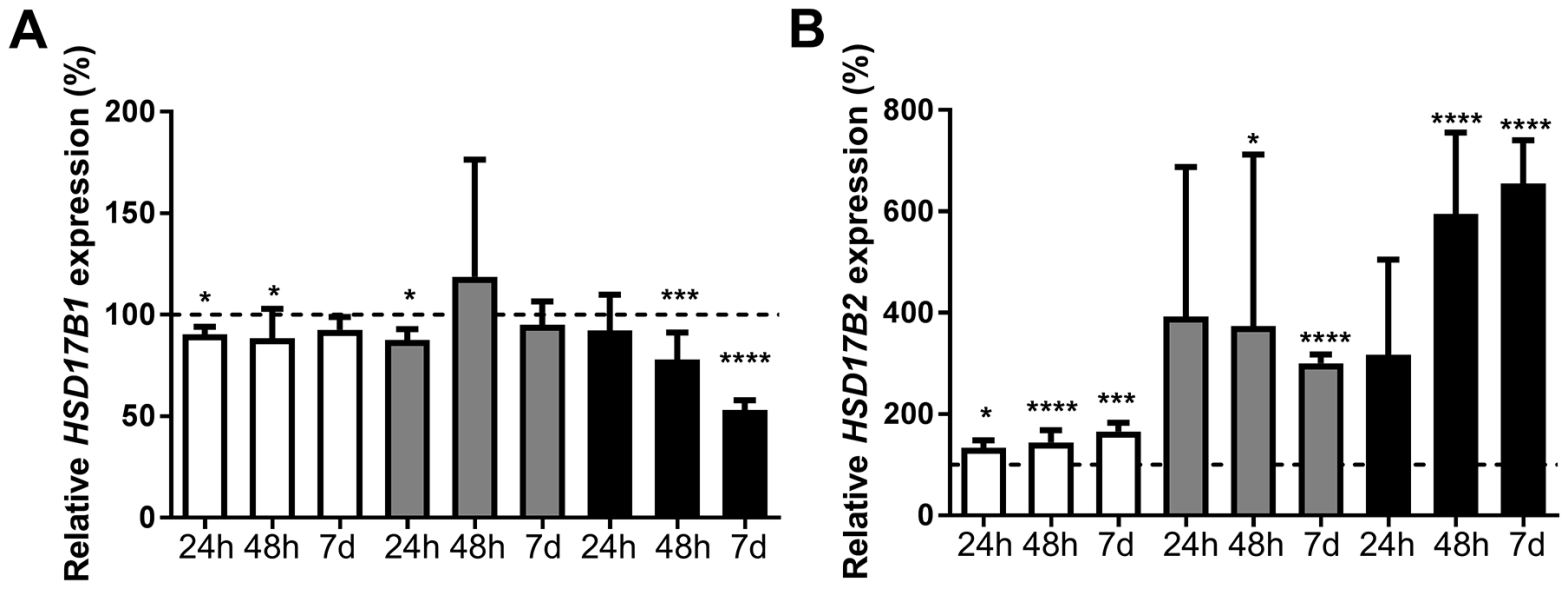
Relative HSD17B1 **(A)** and HSD17B2 **(B)** expression following dihydrotestosterone treatment in ZR-75-1 (white), MCF7 (gray) and T-47D (black), for 24h n=3, 48h n=6, and 7 days n=5. All effects on the relative HSD17B1 and HSD17B2 expression are compared to ethanol-treated controls from the same time point and cell line as the treated sample. Error bars represent standard derivation.

ZR-75-1, MCF7, and T-47D were treated with 10nM dihydrotestosterone for 6 hours, 24 hours, 48 hours, or 7 days. Expression of *HSD17B1* and *HSD17B2* was compared with ethanol-treated controls at the corresponding time point. At 6 hours, there was no significant change in terms of *HSD17B1* expression, but a trend towards an increase in *HSD17B2* expression in all the cell lines (data not shown). In ZR-75-1 cells the expression of *HSD17B1* was significantly decreased to 90% at 24 hours and 88% at 48 hours. In MCF7 cells there was a decrease to 88% at 24 hours. For T-47D cells there was a reduction to 78% at 48 hours and 53% at 7 days, Figure 3A. In ZR-75-1 the *HSD17B2* expression was 133% at 24 hours, 145% at 48 hours and 166% at 7 days. For MCF7 cells, there was an increase to 374% at 48 hours and 300% at 7 days. In T-47D cells there was an increase to 595% at 48 hours and 655% at 7 days, Figure 3B. In summary, dihydrotestosterone had a minor effect on HSD17B1 in ZR-75-1, and MCF-7, with a more sizable reduction in T-47D. Further, dihydrotestosterone resulted in an increased *HSD17B2* expression in all cell lines after 48 hours and 7 days.

**Figure 4:**
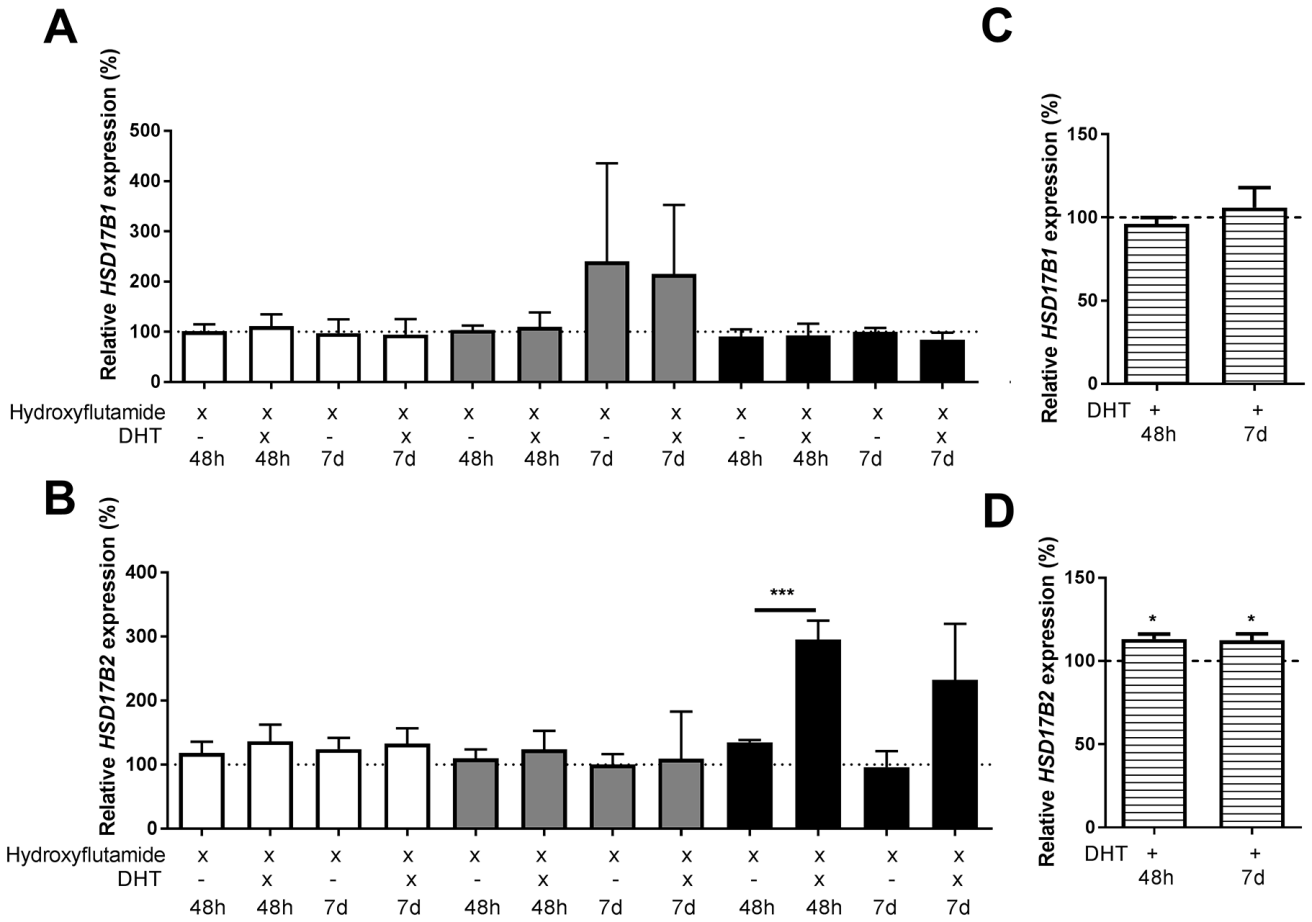
Relative HSD17B1 **(A, C)** and HSD17B2 **(B, D)** expression. (A, B) denotes the effects of dihydrotestosterone in hydroxyflutamide treated ZR-75-1 (white), MCF7 (gray), and T-47D (black), at 48 hours and 7 days, n=3. (C, D) denotes the effect of dihydrotestosterone in SK-BR-3 (horizontal stripes), n=3. All effects of dihydrotestosterone on the relative HSD17B1 and HSD17B2 expression are compared to ethanol-treated controls from the same time point and cell line as the treated sample. Error bars represent standard derivation.

There was no effect on the *HSD17B1* expression following treatment using AR inhibitor hydroxyflutamide in combination with dihydrotestosterone compared to hydroxyflutamide treated control, Figure 4A. Regarding *HSD17B2*, in ZR-75-1 or MCF-7, there was no effect of dihydrotestosterone in combination with hydroxyflutamide compared to hydroxyflutamide treated control. In T-47D, there was an increase in *HSD17B2* to 295% at 48 hours and a similar trend at 7 days, Figure 4B. In the AR-negative cell-line SK-BR-3 there was no effect of dihydrotestosterone on HSD17B1, Figure 4C. Regarding *HSD17B2* there was an increase to 113% at 48 hours and 112% at 7 days, Figure 4D.

### MicroRNA control of HSD17B1 and HSD17B2

**Figure 5:**
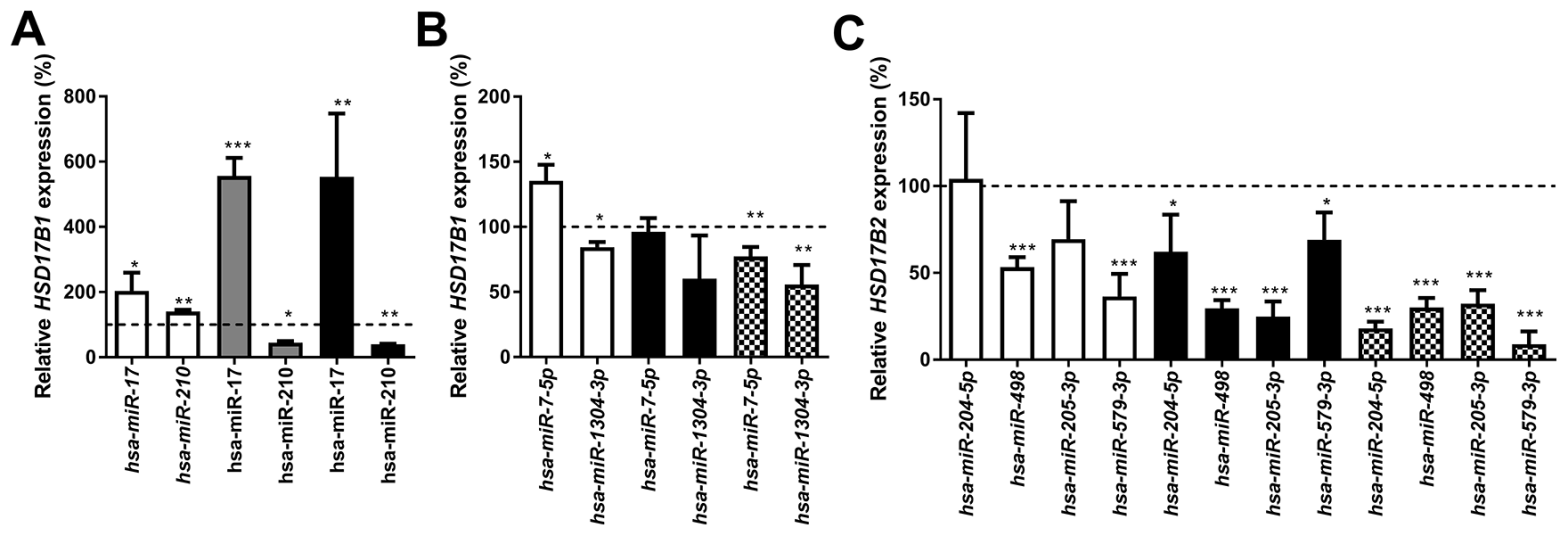
Relative HSD17B1 **(A, B)** and HSD17B2 **(C)** following miRNA treatment for 48 hours. The effects of selected miRNA compared to scrambled miRNA control in the same cell line is shown in ZR-75-1 (white), MCF7 (gray), T-47D (black), and MCF10A (Checkered). n=3. Error bars represent standard derivation.

The miRNAs control of the expression of HSD17B1 or HSD17B2 in breast cancer cells was examined using 50 miRNAs selected as described in the methods section. These miRNAs were transiently transfected into ZR-75-1, MCF7, T-47D and MCF10A cells. Based on preliminary findings *miR-7-5p*, *miR-17*, *miR-210*, *miR-518c*, *miR-1304-3p*, *miR-204-5p*, *miR-205-3p*, *miR-498* and *miR-579-3p* were selected for further study. All experiments were compared with a negative miRNA control. Treatment with *miR-17* resulted in upregulation of *HSD17B1* to 203%, 556% and 554% in ZR-75-1, MCF7 and T-47D cells respectively. Additionally, *miR-210* resulted in 140%, 45% and 40% *HSD17B1* expression in ZR-75-1, MCF7 and T-47D cells respectively, Figure 5A. Following *miR-7-5p* treatment, *HSD17B1* expression was 135% and 77% in ZR-75-1 and MCF10A cells respectively, with no change in T-47D cells. Further, *miR-1304-3p* resulted in 85% and 66% *HSD17B1* expression in ZR-75-1 and MCF10A cells respectively, Figure 5B. Treatment with *miR-498* resulted in 53%, 30% and 30% HSD17B2 expression in ZR-75-1, T-47D and MCF10A cells respectively. In addition, *miR-579-3p* treated cells showed 36%, 69% and 9% *HSD17B2* expression in ZR-75-1, T-47D and MCF10A cells respectively. Following *miR-204-5p* treatment, *HSD17B2* expression was 62% and 18% in T-47D and MCF10A cells respectively. Moreover, *miR-205-3p* treatment resulted in 25% and 32% *HSD17B2* expression in T-47D and MCF10A cells, Figure 5C. In summary, *miR-17* resulted in up-regulation of *HSD17B1*, while *miR-210* and *miR-7-5p* resulted in a mixed response and *miR-1304-3p* resulted in reduced *HSD17B1* expression. *MiR-204-5p, 498, 205-3p* and *579-3p* resulted in a reduction of *HSD17B2*, albeit to differing degrees.

### Genes that modulate the expression of HSD17B1 and HSD17B2

**Figure 6:**
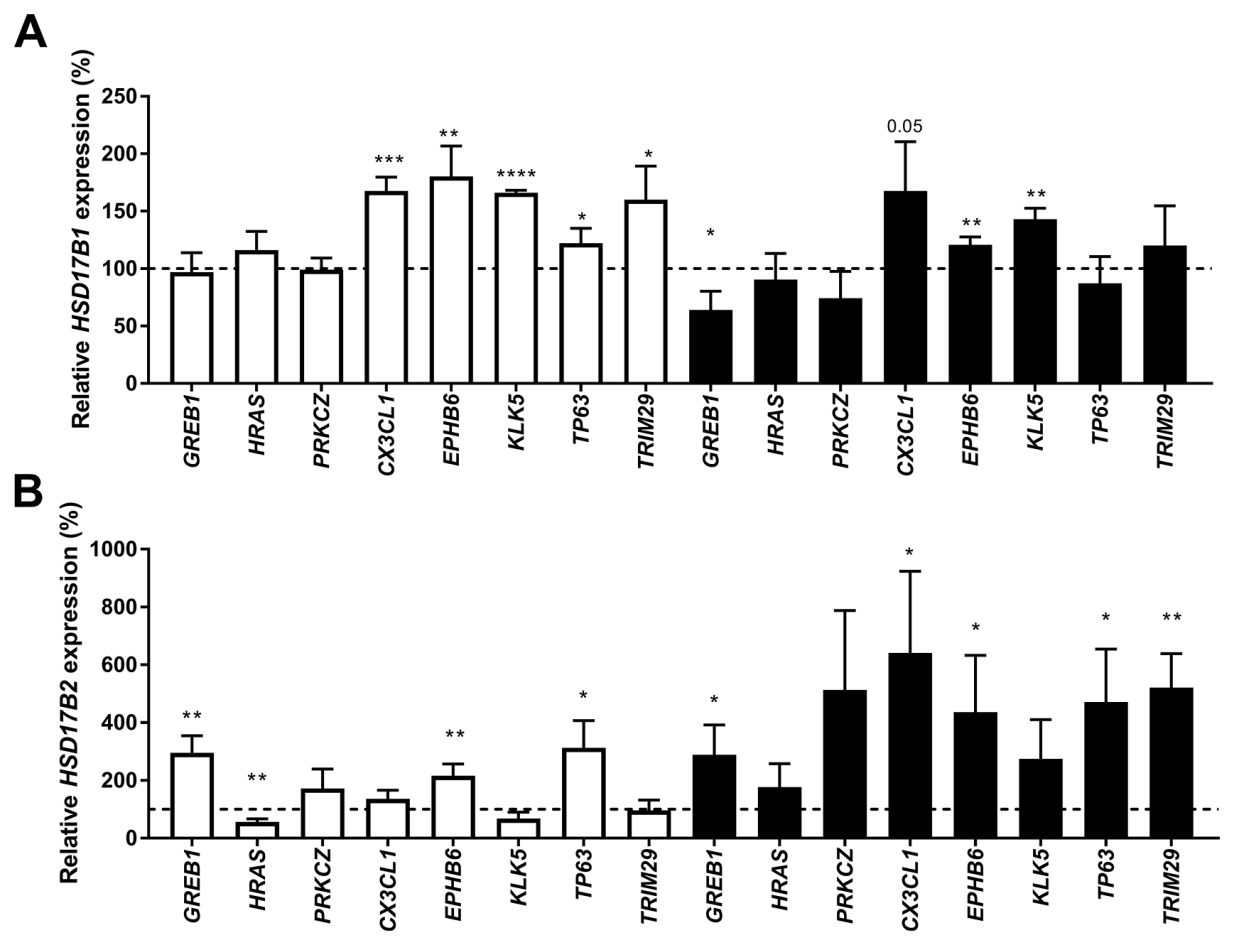
Relative HSD17B1 **(A)** and HSD17B2 **(B)** expression following downregulation of GREB1, HRAS, PRKCZ, CX3CL1, EPHB6, KLK5, TP63 or TRIM29 in ZR-75-1 (white) and T-47D (black) compared to scrambled siRNA from the same cell line, n=3. Error bars represent standard derivation.

We transfected ZR-75-1, MCF7 and T-47D cells with two siRNAs targeting each of the 13 genes strongly correlated to *HSD17B1* or *HSD17B2* and predicted to be involved in breast cancer*.* Based on these findings (data not shown), Growth Regulation By Estrogen In Breast Cancer 1 *(GREB1),* Harvey Rat Sarcoma Viral Oncogene Homolog *(HRAS),* Protein kinase C Zeta *(PRKCZ),* Chemokine (C-X3-C Motif) Ligand 1 *(CX3CL1),* EPH Receptor B6 *(EPHB6),* Kallikrein-Related Peptidase 5 *(KLK5),* Tumor Protein P63 *(TP63),* and Tripartite Motif Containing 29 *(TRIM29)* were chosen for further study. All *HSD17B1* and *HSD17B2* expression were compared to cells treated with scrambled siRNA. In ZR-75-1 cells the *HSD17B1* expression was increased to 168%, 180%, 166%, 122% and 160% when *CX3CL1, EPHB6, KLK5, TP63*, or *TRIM29* were downregulated, respectively. No effect was seen in MCF7 (data not shown). In T-47D cells, *HSD17B1* expression was decreased to 66% in *GREB1* suppressed cells, while HSD17B1 expression was 121% and 143% following *EPHB6*, and *KLK5* downregulation respectively. Additionally, there was a trend towards an increase in *HSD17B1* following *CX3CL1* downregulation in T-47D cells (p=0.052), Figure 6A. In ZR 75-1 cells *HSD17B2* expression was increased to 292%, 216%, and 312% after *GREB1, EPBH6,* and *TP63* downregulation respectively, meanwhile in *HRAS* downregulated ZR-75-1 cells the expression was reduced to 56%. In MCF7 cells there was no effect. In T-47D cells the *HSD17B2* expression was 289%, 641%, 437%, 471% and 521% in *GREB1, CX3CL1*, *EPBH6*, *TP63* and *TRIM29* downregulation respectively, Figure 6B. In summary, GREB1 promotes HSD17B1 expression in T47D and suppresses HSD17B2 in ZR75-1 and T47D. Meanwhile, EPHB6 and KLK6 downregulate HSD17B1 in ZR75-1 and T47D, while EPHB6 and TP63 downregulates HSD17B2 in ZR75-1 and T47D.

## DISCUSSION

Here we demonstrate that estradiol alters the expression of *HSD17B1* and *HSD17B2*, two of the enzymes responsible for mediating the activity of estradiol in breast cancer cell lines. ZR-75-1, MCF7, and T-47D cells are all ERα-positive and represent commonly used cell lines for studying ERα-positive breast cancer. Previously there have been studies where estradiol has been shown to mediate changes in *HSD17B1* expression in lymphocytes [28], but to our knowledge, no such findings have been presented in breast cancer cell lines. In our experiments, the estradiol mediated downregulation of *HSD17B1* was detectable after 24 hours and was further decreased after 48 hours and 7 days, suggesting an increasing effect based on prolonged exposure. In support of this, aromatase inhibitor treatment, which results in estradiol depletion, resulted in increased HSD17B1 expression [29]. Taken together, estradiol mediates negative feedback control of HSD17B1 in ERα-positive breast cancer cell lines, which could be an important mechanism of how local estradiol concentrations are controlled. For *HSD17B2* there was a reduction in response to estradiol at the earlier time points (24 and 48 hours) in both ZR-75-1 and T-47D. However, at 7 days, this reverted to baseline in ZR-75-1 cells but not in T-47D. This suggests that different cell-lines have differing control mechanisms. In MCF7 cells, the *HSD17B2* expression which was unaltered at early time points increased fivefold compared with control after 7 days. Taken together, estradiol control of *HSD17B2* may be time sensitive in cell lines. The increased HSD17B2 expression after 7 days may constitute a mechanism to reduce the estradiol effects after prolonged exposure. In ERα positive cells, ERα downregulation by siRNA prevents the effect of estradiol on *HSD17B1* and *HSD17B2* expression at 48 hours and to some degree at 7 days in ZR-75-1, MCF7 and T-47D cells. Further, in the ERα negative SK-BR-3 cells no effect of estradiol stimulation is detected on *HSD17B1* or *HSD17B2* expression*.* Based on these findings we propose that the *HSD17B1* and *HSD17B2* modulation mediated by estradiol is ERα-dependent. Previous studies showed a correlation between both *HSD17B1* and *HSD17B2* to ERα expression, which supports our findings [19, 22, 24]. Collectively, these findings indicate that ERα-dependent estradiol signaling in breast cancer cell lines results in a reduction in HSD17B1, with a possible time-dependent effect on HSD17B2.

We tested the effect of dihydrotestosterone on both *HSD17B1* and *HSD17B2* expression in the ERα- and AR-positive cell lines ZR-75-1, MCF7, and T-47D. Dihydrotestosterone resulted in a small but significant reduction of *HSD17B1* expression in ZR-75-1 at 24 hours and 48 hours. The reduction in MCF7 was lost after 24 hours. In T47D there was a reduction at 48 hours and 7 days. Regarding *HSD17B2* expression, the expression was markedly increased in all tested cell lines at 48 hours and 7 days. Our results suggest that dihydrotestosterone promotes a more normal-like phenotype of the tissue, reducing *HSD17B1* and increasing *HSD17B2* [30, 31]. Earlier studies conducted in our lab and others show that patients with low HSD17B1 to HSD17B2 ratio have a better prognosis than patients with a higher HSD17B1 to HSD17B2 ratio [21-23]. Furthermore, a low HSD17B1 to HSD17B2 ratio is a positive tamoxifen treatment predictive marker [19]. Taken together, dihydrotestosterone promotes a phenotype more resembling healthy tissue, by increasing the HSD17B2 to HSD17B1 ratio, which would protect the tissue against the effects of estrogens and maintain the dihydrotestosterone concentration. Since we detected no change in HSD17B1 in the hydroxyflutamide treated cells, or in the AR-negative SK-BR-3, this effect seems AR-dependent. HSD17B2 expression changes were greatly reduced following hydroxyflutamide treatment, and no effect of dihydrotestosterone was observed in ZR-75-1 or MCF-7. The persistent effect of dihydrotestosterone on T-47D combined with the small but sizable increase in HSD17B2 expression in SK-BR-3, suggests that AR is not the only component in mediating dihydrotestosterone driven changes in HSD17B2. The presence of ERβ1 [32], which may be activated by 3α- and 3β-diol [33], may be an explanation for this, but further evaluation is needed.

We analyzed the efficiency of miRNA in modulating the mRNA expression of *HSD17B1* and *HSD17B2* based on previously literature and bioinformatics predictions. The primary work in the field of miRNA and HSD17B1 or HSD17B2 to date is a publication showing that *miR-210* and *miR-518c* control the expression of *HSD17B1* in placental tissue [27]. We were unable to see any *HSD17B1* modulating ability from *miR-518c* (data not shown). The role of *miR-210* is oncogenic, being involved in hypoxia, metastasis, and proliferation [34]. We show that m*iR-210* upregulated *HSD17B1* in highly proliferative ZR-75-1 cells and downregulated *HSD17B1* in more slowly proliferating MCF7 and T-47D cells. There are reported cases where miRNA can increase target expression [35]. However, the effect seen could also be an indirect effect of *miR-210* on genes which in turn control *HSD17B1*. The role of *miR-17* appears to differ somewhat between different studies, either promoting apoptosis via increased p53 [36] or promoting invasion and migration via HBP1/β-catenin [37]. Further, it has been associated with the more aggressive triple negative breast cancer subtype [37, 38]. Our findings that *miR-17* increases *HSD17B1* expression would support the oncogenic role of *miR-17*. Regarding *miR-7-5p*, we saw a highly variable response, which could indicate an indirect, or relatively modest direct effect on *HSD17B1*. These findings make us question the relevance of *miR-7-5p* regarding *HSD17B1* regulation. The relatively uncharacterized *miR-1304-3p* caused repression of *HSD17B1* in ZR-75-1 and MCF10A cells, with a similar trend in T-47D cells. Based on these findings *miR-1304-3p* could be relevant in the control of estrogen activity in breast cancer. *MiR-498* is frequently downregulated in several forms of cancer, and we show downregulation of *HSD17B2* upon treatment with *miR-498,* suggesting a pro-estrogenic role of this miRNA*.* Transfection with the tumor suppressors *miR-204-5p* and *miR-205-3p,* [39-42], resulted in decreased *HSD17B2* in T-47D and MCF10A. These findings were surprising since HSD17B2 is tissue protective, the expected effect would have been either no effect or an increase in HSD17B2 following treatment. Lastly, we show that the poorly characterized *miR-579-3p* is a potent down regulator of *HSD17B2* in ZR-75-1 and MCF10A, providing some of the first hints as to its role in breast cancer. Taken together, these findings are the first to characterize miRNAs controlling HSD17B1 and HSD17B2 in breast cancer cell lines. Further knowledge of the expression patterns of these miRNAs in breast cancer is still needed.

To determine if other genes involved in breast cancer could mediate changes in *HSD17B1* or *HSD17B2* expression, we analyzed the effect of downregulation of *GREB1, HRAS, PRKCZ, CX3CL1, EPHB6, KLK5, TP63,* and *TRIM29* on *HSD17B1* and *HSD17B2* gene expression. CX3CL1, EPHB6, KLK5, GREB1, and TP63 are all associated with adverse outcome when expressed [43-56]. A more detailed list of the function of these genes is found in Supplementary Table 4 [43-56]. The estrogen response gene *GREB1* seemed to promote *HSD17B1* and suppress *HSD17B2* in T47D cells, which would promote increased steroid activation. Also, we found that upon *CX3CL1*, *EPHB6,* and *KLK5* downregulation, HSD17B1 expression increased. Downregulation of GREB1, *EPBH6* and *TP63* resulted in increased HSD17B2 expression. There was a suppressive effect of CX3CL1 and TP63 on both HSD17B1 and HSD17B2. These findings could indicate a switch to a more steroid independent phenotype as the tumor progresses, and steroid activity is less pivotal in determining the future of the tumor.

We used the mRNA expression to determine the effect of steroids, miRNA and the genes involved in breast cancer cell lines, which is a reliable and robust method to detected changes in gene expression, with the drawback that mRNA expression does not always correlate to protein expression. Further, we used mRNA to quantify the effect of the miRNA. While an efficient approach, it does have the drawback of not being able to detect partial binding of mRNA which does not result in degradation of the mRNA target. However, expression analyses of miRNA targets are unable to detect if miRNA effects are direct or indirect, which makes the determination of miRNA true targets more difficult.

## MATERIALS AND METHODS

### Cell lines

The breast cancer epithelial cell lines ZR-75-1, MCF7, T-47D, and SK-BR-3, as well as the immortalized epithelial cell line MCF10A were acquired from the American Type Culture Collection (ATCC, Manassas, VA). The authenticity of the cell lines was validated using short-tandem repeat (STR)s profiling prior to purchase. All experiments were conducted while cells were in passages 5-30. ZR-75-1, MCF7, and T-47D cells are ERα and AR-positive, Supplementary Figure 3, and express HSD17B1 and HSD17B2. SK-BR-3 and MCF10A are ERα and AR-negative but express HSD17B1 and HSD17B2. ZR-75-1, MCF7, T-47D cells, and SK-BR-3 were maintained in OptiMem (Thermo Fisher Scientific, MA, USA) without phenol red supplemented with 4% (v/v) FBS. MCF10A was maintained in Mammary Epithelial Cell Growth Medium (MEGM) w/o phenol red supplemented with bovine pituitary extract, hydrocortisone, human epithelial growth factor and insulin (Lonza, Basel, Switzerland) and 100ng/ml cholera toxin (C8052, Merck KGaA, Darmstadt Germany). All cells were maintained at 5% CO_2_ at 37°C in a humidified incubator.

### Hormonal treatment

ZR-75-1, MCF7, T-47D, and SK-BR-3 cells were seeded in media containing FBS. Eight hours after seeding, the cells were switched to charcoal treated serum (CTS) and incubated for 16 hours before addition of hormone treatment. Estradiol (E8875, Merck) and dihydrotestosterone (A8380, Merck) were diluted in 99.5% ethanol and added to CTS containing media for a final ethanol concentration of 0.1% (v/v) 5-10 minutes before addition to the cells, and the medium was replaced every 24 hours. Final estradiol concentration was 5nM or 10nM and dihydrotestosterone concentration was 10nM. Cells were harvested after 24, 48 hours or 7 days of steroid treatment. All treatments were done in triplicates and repeated three to six times.

### Treatment with ERα siRNA and Hormonal treatment

ZR-75-1, MCF7, and T-47D were transfected using reverse transfection of 11.25nM estrogen receptor siRNA s4823 (Thermo Fisher Scientific) using Dharmafect 1 with a final concentration of 0.5% (v/v) out of the total media volume (GE Healthcare, Little Chalfont, United Kingdom) and incubated for 30 hours. At this time the media was changed to CTS containing media. After a 16-hour incubation in CTS, freshly prepared 5nM estradiol containing CTS medium was added to the cells and subsequently changed every 24 hours. Cells were harvested 48 hours or 7 days after the beginning of the estradiol treatment. All treatments were done in triplicates and repeated three times.

### Treatment with AR inhibitor

ZR-75-1, MCF7, and T-47D cells were seeded in media containing FBS. Eight hours after seeding, the cells were switched to CTS and incubated for 16 hours before addition of hormone treatment. Dihydrotestosterone (A8380, Merck) was diluted in 99.5% ethanol. Hydroxyflutamide (H4166, Merck) was diluted in DMSO. dihydrotestosterone and hydroxyflutamide were added to CTS containing media for a final ethanol and DMSO concentration of 0.1% (v/v) 5-10 minutes before addition to the cells, and the medium was replaced every 24 hours. Final dihydrotestosterone concentration was 10nM, and the final hydroxyflutamide concentration was 10μM. Cells were harvested 48 hours or 7 days after the start of steroid treatment. All treatments were done in triplicates and repeated three times.

### Treatment with miRNA

The miRNAs were chosen based on a literature study and bioinformatics predictions using miRSearch v3.0 (exiqon), microT v4 [57] and TargetScan [58]. All miRNA experiments were carried out using miRIDIAN microRNA mimics (GE Healthcare) or mirVana miRNA mimics (Thermo Fisher Scientific). A full list of miRNAs used is shown in Supplementary Table 1. ZR-75-1, MCF7, T-47D, and MCF10A cells were seeded 24 hours prior to transfection. The microRNA mimic was added at a final concentration of 40nM combined with Dharmafect 1 (GE Healthcare) with a final concentration of 0.5% (v/v) out of the total media volume. The cells were harvested at 48 hours after transfection. An initial screening to determine which miRNA efficiently regulated *HSD17B1* or *HSD17B2* was performed two times. The experiments to validate miRNA presented in this work were performed in triplicates and repeated three times.

### Treatment with siRNA

We prioritized genes for this study by their co-expression with HSD17B1 and HSD17B2 using published transcriptomics datasets (Cancer Genome Atlas Network, 2012). The whole TCGA breast cancer cohort included 1098 patients. We analyzed the co-expression of HSD17B1 and HSD17B2 with protein-coding genes only in the ERα-positive samples. The ERα-positive parts of the transcriptomics datasets from Agilent microarrays, as well as RNA-sequencing of versions 1 and 2, included 430, 630, and 830 patients, respectively. The strength and significance of the correlation between either HSD17B1 or HSD17B2 and any other genes *(G)* were analyzed in a multiple regression models, which also included available clinical covariates known to affect the breast cancer transcriptome, namely the AJCC pathologic tumor stage, progesterone receptor status, and HER2 status:

$$E_{HSD17B\left(1,2\right)}=\beta_{1}C_{AJCC}+\beta_{2}C_{PR}+\beta_{3}C_{HER2}+\beta_{4}E_{G}+\varepsilon$$

In this framework, the p-values for the *β*_*4*_*E*_*G*_ term reported net correlations between gene expression profiles of HSD17B and *G*, i.e. independently of the AJCC, PR, and HER2 values. The p-value sets from each transcriptomics dataset were separately adjusted for multiple testing by Benjamini and Hochberg [59]. In parallel, we calculated Spearman rank correlation coefficients between HSD17B (1, 2) and *G.* By requiring absolute rank r>0.35 and adjusted p *(β*_*4*_*E*_*G*_) <0.01, we obtained a list of 382 distinct genes. The results from all the transcriptomics datasets together were ranked by p-value and then the top 80 genes were subjected to a literature search of possible connections of these genes to estrogen or androgen signaling in breast cancer (keywords used were breast cancer, estrogen, Estrogen receptor, proliferation, AKT, MAPK, migration, metastasis, PI3K, tumor invasion, androgens, aromatase). Finally, the 13 most promising genes were selected for down-regulation by siRNA. Two siRNAs were transfected for each gene. A full list of siRNA is shown in Supplementary Table 2. ZR-75-1, MCF7, and T-47D cells were transfected using reverse transfection of 10nM silence select siRNA (Thermo Fisher Scientific) using Dharmafect 1 (GE Healthcare) with a final concentration of 0.5% (v/v) out of the total media volume. The cells were harvested at 48 hours after transfection. The initial screening was performed twice. Based on the screening, *GREB1, HRAS, PRKCZ, CX3CL1, EPHB6, KLK5, TP63,* and *TRIM29* were chosen for validation. The validation experiments were performed in triplicates and repeated thrice.

### Semi-quantitative polymerase chain reaction (qPCR)

RNA from the hormonal treatment, microRNA, and siRNA experiments was isolated using TRIzol (Thermo Fisher Scientific) according to manufacturer’s instructions. Reverse transcription-PCR was performed using the High Capacity cDNA kit (Thermo Fisher Scientific). The qPCR reaction was conducted using TaqMan® Fast Universal PCR Master Mix without AmpErase UNG (Thermo Fisher Scientific). A list of assays used can be found in Supplementary Table 3. For *HSD17B1* the following custom primer/probes were used: forward primer 5’-TAT GCG AGA GTC TGG CGG TT-3’, reverse primer; 5’-TGC ACT GGG CCG CAC T-3’ and the probe 5’-CGA TCA GGC TCA AGT GGA CCC CAA-3’. For all experiments Peptidylprolyl Isomerase A (Cyclophilin A), and beta-actin were used as endogenous controls. Data analyses were performed according to the ΔΔCt method, and relative concentrations were calculated against the appropriate control, detailed in the respective result section.

### Statistical analysis

The differences between groups were analyzed using Student’s t-test. GraphPad Prism 7 (GraphPad Software, CA, USA) was used for all calculations and constructing of graphs. P-value cutoff was set at 0.05. In the figures, the following p-value symbols are used; * p<0.05, ** p<0.01 *** p<0.001 **** p<0.0001. Numbers in the graphs denote non-significant p-values.

## CONCLUSION

In conclusion, we show that estradiol negatively regulates *HSD17B1* in breast cancer cell lines, and a possible time sensitive modulator of HSD17B2. This effect appears to be reliant on ERα since no response was seen in ERα negative cells or cells treated with siRNA against ERα at 48 hours. Further, dihydrotestosterone has a primarily reductive impact on HSD17B1 expression, but the primary effect seen was an increase in HSD17B2 expression in all tested cell lines. This appears to be reliant on at least in part signaling through AR since a minor response was seen in AR-negative cells or and a reduced response was seen in cells treated with hydroxyflutamide. We found eight miRNAs which modulate HSD17B1 or HSD17B2 expression, and five genes which appear able to control HSD17B1 or HSD17B2 expression in breast cancer cell lines. These findings shed light on the complex control mechanisms for how the two primary steroid converting HSD17 enzymes are regulated in breast cancer cell lines, and will hopefully open up for further lines of study which could lead to therapeutic interventions.

## SUPPLEMENTARY MATERIALS FIGURES AND TABLES







## Abbreviations

ER: estrogen receptor
AR: androgen receptor
STS: steroid sulfatase
DHEA: dehydroepiandrosterone
miR: microRNA
*GREB1*: Growth Regulation By Estrogen In Breast Cancer 1
*HRAS*: Harvey Rat Sarcoma Viral Oncogene Homolog
PRKCZ: Protein kinase C Zeta
*CX3CL1*: Chemokine (C-X3-C Motif) Ligand 1
EPHB6: EPH Receptor B6
KLK5: Kallikrein-Related Peptidase 5
TP63: Tumor Protein P63
TRIM29: Tripartite Motif Containing 29
