# Supplementary material for: The regulation of hydroxysteroid 17β-dehydrogenase type 1 and 2 gene expression in breast cancer cell lines by estradiol, dihydrotestosterone, microRNAs, and genes related to breast cancer
Source: Oncotarget. 2017 Jul 10;8(37):62183–94. doi: 10.18632/oncotarget.19136 (PMC5617496; doi:10.18632/oncotarget.19136)
Supplement: Supplementary file 1 [file oncotarget-08-62183-s001.pdf]

The regulation of hydroxysteroid 17β-dehydrogenase type 1 and 2 gene expression in breast cancer cell lines by estradiol, dihydrotestosterone, microRNAs, and genes related to breast cancer

SUPPLEMENTARY MATERIALS

Protein blotting

Protein from the hormonal treatment was isolated using RIPA, followed by centrifugation at 12 000g for 10 minutes. Protein lysates (25µg) were mixed 1:1 with Laemmli sample buffer (Bio-Rad, Hercules, CA, USA) heated in 95°C and loaded onto Mini-PROTEAN TGX precast gels (4-15% Tris-HCl; Bio-Rad). The proteins were separated at 90 V for 5 min followed by 150 V for 35 minutes using 1x Tris-Glycine-SDS (TGS) buffer, transferred onto a PVDF membrane (TBT Blotting System Transfer Pack; Bio-Rad) in a Trans-Blot® TurboTM (Bio-Rad). The membranes were washed in Tris-buffered Saline (TBS) + 0.1% tween20, and blocked in TBS + 0.1% tween20 (TBST) + 5% non-fat powder milk for 60 min in room temperature. The primary antibody monoclonal rabbit anti-human estrogen receptor (04-820, Clone 60c, Merck KGaA, Darmstadt Germany) was diluted 1:1000 in TBST 5% milk, and incubated in 4°C overnight. The membranes were washed and incubated for an hour with the secondary antibody (1:1 000, anti-rabbit; DAKO) in room temperature. The binding of the antibody to the membrane was detected using ELCprime + Western Blotting Detection System (Amersham Biosciences/ GE Healthcare, Piscataway, NJ, USA), and developed on an Amersham HyperfilmTM ECL (GE Healthcare, Piscataway, NJ, USA). GAPDH 1:5000 (EPR6256 Abcam, Cambridge, UK) was used as a reference protein.

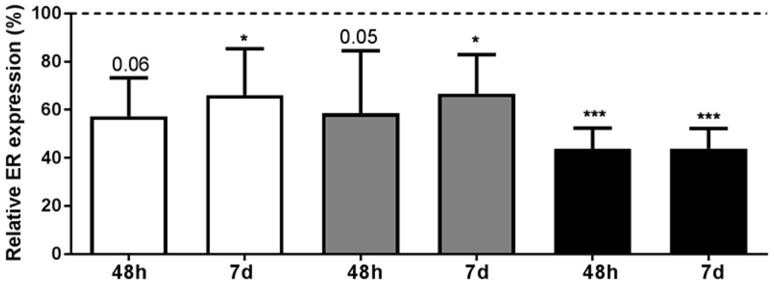

Supplementary Figure 1: Relative ERα expression following ERα downregulation at 48 hours and 7 days compared to scrambled siRNA control. ZR-75-1 (white), MCF7 (gray) and T-47D (black), n=3. Error bars represent standard derivation.

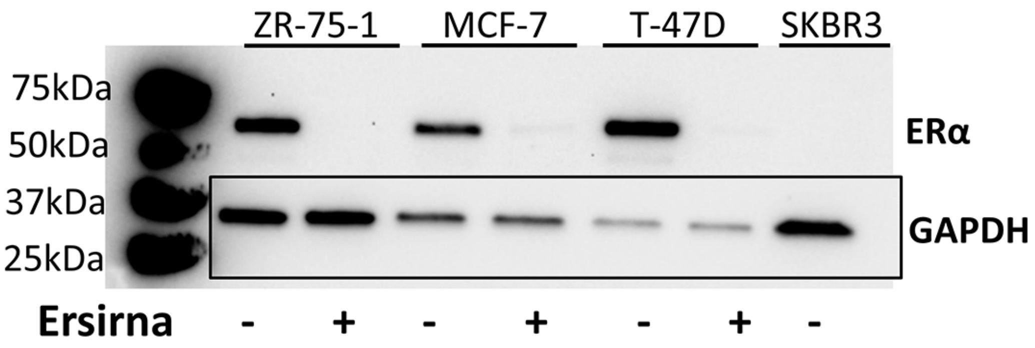

Supplementary Figure 2: Protein expression of ERα at 48 hours (04-820, Clone 60c, Merck KGaA) following ER siRNA or scrambled siRNA respectively in ZR-75-1 (1-2), MCF7 (3-4), T-47D (5-6), and untreated SK-BR-3 (7). GAPDH (EPR6256 Abcam, Cambridge, UK) was used as a reference protein. All bands were exposed for the same duration and treated the same in image generation and no modifications were performed apart from image cropping. See supplement materials and methods for details of the experimental conditions.

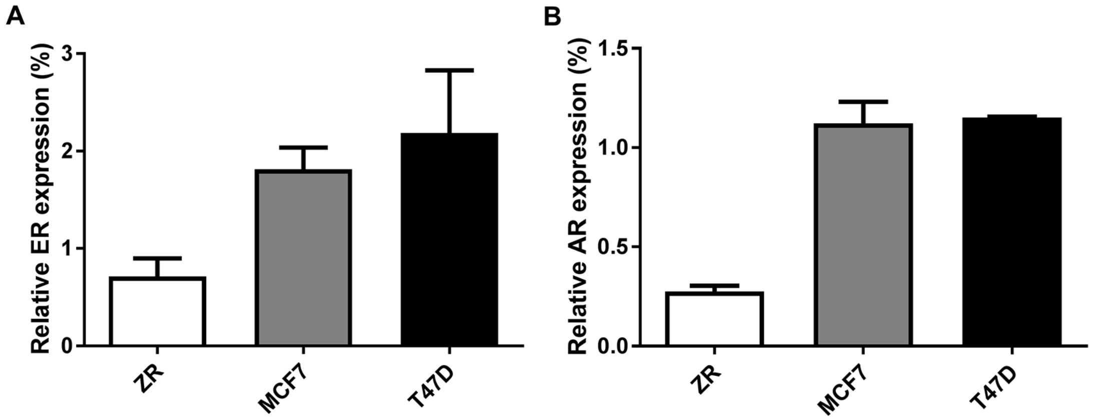

Supplementary Figure 3: The relative amount of ERα and AR at 48 hours. ZR-75-1 (white), MCF7 (gray) and T-47D (black), n=3. Error bars represent standard derivation.

Supplementary Table 1: miRNA used in the current study

See Supplementary File 1

Supplementary Table 2: siRNA used in the current study

GREB1	SilenceSelect siRNA	Thermo Fisher Scienticic, MA, USA	s18650
**GREB1**	**SilenceSelect siRNA**	**Thermo Fisher Scienticic, MA, USA**	**s18651**
**HRAS**	**SilenceSelect siRNA**	**Thermo Fisher Scienticic, MA, USA**	**s808**
HRAS	SilenceSelect siRNA	Thermo Fisher Scienticic, MA, USA	s807
**PRKCZ**	**SilenceSelect siRNA**	**Thermo Fisher Scienticic, MA, USA**	**s11128**
PRKCZ	SilenceSelect siRNA	Thermo Fisher Scienticic, MA, USA	s11129
ACE2	SilenceSelect siRNA	Thermo Fisher Scienticic, MA, USA	s33966
ACE2	SilenceSelect siRNA	Thermo Fisher Scienticic, MA, USA	s33965
CHI3L1/YKL40	SilenceSelect siRNA	Thermo Fisher Scienticic, MA, USA	s2999
CHI3L1/YKL40	SilenceSelect siRNA	Thermo Fisher Scienticic, MA, USA	s2998
CX3CL1	SilenceSelect siRNA	Thermo Fisher Scienticic, MA, USA	s226987
**CX3CL1**	**SilenceSelect siRNA**	**Thermo Fisher Scienticic, MA, USA**	**s12631**
**ER**	**SilenceSelect siRNA**	**Thermo Fisher Scienticic, MA, USA**	**S4823**
**EPHB6**	**SilenceSelect siRNA**	**Thermo Fisher Scienticic, MA, USA**	**s4748**
EPHB6	SilenceSelect siRNA	Thermo Fisher Scienticic, MA, USA	s4747
KLK5	SilenceSelect siRNA	Thermo Fisher Scienticic, MA, USA	s223657
**KLK5**	**SilenceSelect siRNA**	**Thermo Fisher Scienticic, MA, USA**	**s223656**
KLK7	SilenceSelect siRNA	Thermo Fisher Scienticic, MA, USA	s11269
KLK7	SilenceSelect siRNA	Thermo Fisher Scienticic, MA, USA	s11268
OLFM4	SilenceSelect siRNA	Thermo Fisher Scienticic, MA, USA	s20724
OLFM4	SilenceSelect siRNA	Thermo Fisher Scienticic, MA, USA	s20722
TP63	SilenceSelect siRNA	Thermo Fisher Scienticic, MA, USA	s229399
**TP63**	**SilenceSelect siRNA**	**Thermo Fisher Scienticic, MA, USA**	**s16411**
**TRIM29**	**SilenceSelect siRNA**	**Thermo Fisher Scienticic, MA, USA**	**s24283**
TRIM29	SilenceSelect siRNA	Thermo Fisher Scienticic, MA, USA	s24285

* Bold siRNA were used for the validation, and results from them are shown in the paper.

Supplementary Table 3: Taqman assays used in the current study

ER	hs00174860_m1	Thermo Fisher Scienticic, MA, USA
HSD17B2	Hs00157993_m1	Thermo Fisher Scienticic, MA, USA
PPiA	Hs99999904_m1	Thermo Fisher Scienticic, MA, USA
BAKT	4352935E	Thermo Fisher Scienticic, MA, USA
GREB1	Hs00536409_m1	Thermo Fisher Scienticic, MA, USA
HRAS	Hs00978050_g1	Thermo Fisher Scienticic, MA, USA
PRKCZ	Hs00177051_m1	Thermo Fisher Scienticic, MA, USA
ACE2	Hs01085333_m1	Thermo Fisher Scienticic, MA, USA
CHI3L1/YKL40	Hs01072228_m1	Thermo Fisher Scienticic, MA, USA
CX3CL1	Hs00171086_m1	Thermo Fisher Scienticic, MA, USA
EPHB6	Hs01071143_g1	Thermo Fisher Scienticic, MA, USA
KLK5	Hs01548153_m1	Thermo Fisher Scienticic, MA, USA
KLK7	Hs00192503_m1	Thermo Fisher Scienticic, MA, USA
OLFM4	Hs04234962_m1	Thermo Fisher Scienticic, MA, USA
TP63	Hs00978340_m1	Thermo Fisher Scienticic, MA, USA
TRIM29	Hs00232590_m1	Thermo Fisher Scienticic, MA, USA
TRPV6	Hs01114089_g1	Thermo Fisher Scienticic, MA, USA

Supplementary Table 4: Roles of the tested genes in the current study

See Supplementary File 2
