# Supplementary material for: The regulation of hydroxysteroid 17β-dehydrogenase type 1 and 2 gene expression in breast cancer cell lines by estradiol, dihydrotestosterone, microRNAs, and genes related to breast cancer
Source: Oncotarget. 2017 Jul 10;8(37):62183–94. doi: 10.18632/oncotarget.19136 (PMC5617496; doi:10.18632/oncotarget.19136)
Supplement: Supplementary file 2 [file oncotarget-08-62183-s002.docx]

| Supplementary Table 1: miRNA used in the current study | | |
| --- | --- | --- |
| ***hsa-miR-7-5p*** | **mirVana** | **Thermo Fisher Scienticic, MA, USA** |
| ***hsa-miR-17*** | **miRIDIAN** | **GE Healthcare, Little Chalfont, United Kingdom** |
| *hsa-miR-22* | miRIDIAN | GE Healthcare, Little Chalfont, United Kingdom |
| *hsa-miR-26b-5p* | mirVana | Thermo Fisher Scienticic, MA, USA |
| *hsa-miR-124-3p* | mirVana | Thermo Fisher Scienticic, MA, USA |
| *hsa-miR-150-5p* | mirVana | Thermo Fisher Scienticic, MA, USA |
| *hsa-miR-181a-5p* | mirVana | Thermo Fisher Scienticic, MA, USA |
| *hsa-miR-181b-5p* | mirVana | Thermo Fisher Scienticic, MA, USA |
| *hsa-miR-181c-5p* | mirVana | Thermo Fisher Scienticic, MA, USA |
| *hsa-miR-181d-5p* | mirVana | Thermo Fisher Scienticic, MA, USA |
| *hsa-miR-198* | mirVana | Thermo Fisher Scienticic, MA, USA |
| ***hsa-miR-204-5p*** | **mirVana** | **Thermo Fisher Scienticic, MA, USA** |
| ***hsa-miR-205-3p*** | **mirVana** | **Thermo Fisher Scienticic, MA, USA** |
| ***hsa-miR-210*** | **miRIDIAN** | **GE Healthcare, Little Chalfont, United Kingdom** |
| *hsa-miR-211-5p* | mirVana | Thermo Fisher Scienticic, MA, USA |
| *hsa-miR-302b-5p* | mirVana | Thermo Fisher Scienticic, MA, USA |
| *hsa-miR-302d-5p* | mirVana | Thermo Fisher Scienticic, MA, USA |
| *hsa-miR-423-3p* | mirVana | Thermo Fisher Scienticic, MA, USA |
| *hsa-miR-494-3p* | mirVana | Thermo Fisher Scienticic, MA, USA |
| ***hsa-miR-498*** | **mirVana** | **Thermo Fisher Scienticic, MA, USA** |
| *hsa-miR-518c* | miRIDIAN | GE Healthcare, Little Chalfont, United Kingdom |
| *hsa-miR-562* | mirVana | Thermo Fisher Scienticic, MA, USA |
| ***hsa-miR-579-3p*** | **mirVana** | **Thermo Fisher Scienticic, MA, USA** |
| *hsa-miR-580-3p* | mirVana | Thermo Fisher Scienticic, MA, USA |
| *hsa-miR-744-5p* | mirVana | Thermo Fisher Scienticic, MA, USA |
| *hsa-miR-873-5p* | mirVana | Thermo Fisher Scienticic, MA, USA |
| *hsa-miR-892a* | mirVana | Thermo Fisher Scienticic, MA, USA |
| *hsa-miR-892b* | mirVana | Thermo Fisher Scienticic, MA, USA |
| *hsa-miR-1273f* | mirVana | Thermo Fisher Scienticic, MA, USA |
| ***hsa-miR-1304-3p*** | **mirVana** | **Thermo Fisher Scienticic, MA, USA** |
| *hsa-miR-1305* | mirVana | Thermo Fisher Scienticic, MA, USA |
| *hsa-miR-1909-3p* | mirVana | Thermo Fisher Scienticic, MA, USA |
| *hsa-miR-1915-3p* | mirVana | Thermo Fisher Scienticic, MA, USA |
| *hsa-miR-2277-5p* | mirVana | Thermo Fisher Scienticic, MA, USA |
| *hsa-miR-3064-5p* | mirVana | Thermo Fisher Scienticic, MA, USA |
| *hsa-miR-3170* | mirVana | Thermo Fisher Scienticic, MA, USA |
| *hsa-miR-3178* | mirVana | Thermo Fisher Scienticic, MA, USA |
| *hsa-miR-3196* | mirVana | Thermo Fisher Scienticic, MA, USA |
| *hsa-miR-3674* | mirVana | Thermo Fisher Scienticic, MA, USA |
| *hsa-miR-4261* | mirVana | Thermo Fisher Scienticic, MA, USA |
| *hsa-miR-4481* | mirVana | Thermo Fisher Scienticic, MA, USA |
| *hsa-miR-4508* | mirVana | Thermo Fisher Scienticic, MA, USA |
| *hsa-miR-4745-5p* | mirVana | Thermo Fisher Scienticic, MA, USA |
| *hsa-miR-4794* | mirVana | Thermo Fisher Scienticic, MA, USA |
| *hsa-miR-5006-3p* | mirVana | Thermo Fisher Scienticic, MA, USA |
| *hsa-miR-5095* | mirVana | Thermo Fisher Scienticic, MA, USA |
| *hsa-miR-5708* | mirVana | Thermo Fisher Scienticic, MA, USA |
| *hsa-miR-6504-5p* | mirVana | Thermo Fisher Scienticic, MA, USA |
| *hsa-miR-6784-5p* | mirVana | Thermo Fisher Scienticic, MA, USA |
| *hsa-miR-6832-5p* | mirVana | Thermo Fisher Scienticic, MA, USA |

***Results from bold miRs are presented in the paper**
