# Supplementary material for: The regulation of hydroxysteroid 17β-dehydrogenase type 1 and 2 gene expression in breast cancer cell lines by estradiol, dihydrotestosterone, microRNAs, and genes related to breast cancer
Source: Oncotarget. 2017 Jul 10;8(37):62183–94. doi: 10.18632/oncotarget.19136 (PMC5617496; doi:10.18632/oncotarget.19136)
Supplement: Supplementary file 3 [file oncotarget-08-62183-s003.docx]

| **Supplementary Table 4: Roles of the tested genes in the current study** | | | |
| --- | --- | --- | --- |
| **Full name** | Short name | Role | Reference |
| Growth Regulation By Estrogen In Breast Cancer 1 | GREB1 | ERα response gene. It has been reported to drive proliferation. Acts as an oncogene. | [1-3] |
| Harvey Rat Sarcoma Viral Oncogene Homolog | HRAS | Involved in PI3K-Akt signaling and MAPK signaling. Oncogene involved in migration and metastasis. Mutations have been implicated in breast cancer prevalence. Downstreams target of ERα. | [4-6] |
| Protein kinase C Zeta | PRKCZ | Facilitates breast cancer invasion and metastasis. | [7, 8] |
| Chemokine (C-X3-C Motif) Ligand 1 | CX3CL1 | Reported to promote immune cell infiltration, and act as a tumor suppressor. In breast cancers with low infiltration, it is associated with worse prognosis and metastasis to the bone. Also reported to drive ErbB mediated proliferation of tumor cells. | [9-11] |
| EPH Receptor B6 | EPHB6 | Primarily adverse outcome, as EPHB6 has been shown to predict poor patient outcome. However, it is frequently down-regulated in progressive breast cancer, and has been reported to suppress oncogenes including other ephrins though miRNA signaling and direct interactions | [12-14] |
| Kallikrein-Related Peptidase 5 | KLK5 | Conflicting results, associated with worse prognosis but frequently down regulated in breast cancer. KLK5 has been shown to suppress metastatic genes. Co-expressed with KLK7. | [15-17] |
| Tumor Protein P63 | TP63 | ERα response gene, promotes proliferation, differentiation and metastasis | [18, 19] |
| Tripartite Motif Containing 29 | TRIM29 | Tumor suppressor which is up-regulated by hypoxia. It reduces metastatic behavior and is associated with improved patient outcome | [20, 21] |

1. Laviolette LA, Hodgkinson KM, Minhas N, Perez-Iratxeta C and Vanderhyden BC. 17beta-estradiol upregulates GREB1 and accelerates ovarian tumor progression in vivo. Int J Cancer. 2014; 135(5):1072-1084.

2. Chand AL, Wijayakumara DD, Knower KC, Herridge KA, Howard TL, Lazarus KA and Clyne CD. The orphan nuclear receptor LRH-1 and ERalpha activate GREB1 expression to induce breast cancer cell proliferation. PLoS One. 2012; 7(2):e31593.

3. Liu M, Wang G, Gomez-Fernandez CR and Guo S. GREB1 functions as a growth promoter and is modulated by IL6/STAT3 in breast cancer. PLoS One. 2012; 7(10):e46410.

4. Holen I, Nutter F, Wilkinson JM, Evans CA, Avgoustou P and Ottewell PD. Human breast cancer bone metastasis in vitro and in vivo: a novel 3D model system for studies of tumour cell-bone cell interactions. Clin Exp Metastasis. 2015; 32(7):689-702.

5. Hall JM, Huey B, Morrow J, Newman B, Lee M, Jones E, Carter C, Buehring GC and King MC. Rare HRAS alleles and susceptibility to human breast cancer. Genomics. 1990; 6(1):188-191.

6. Garrett PA, Hulka BS, Kim YL and Farber RA. HRAS protooncogene polymorphism and breast cancer. Cancer epidemiology, biomarkers & prevention : a publication of the American Association for Cancer Research, cosponsored by the American Society of Preventive Oncology. 1993; 2(2):131-138.

7. Wu J, Liu S, Fan Z, Zhang L, Tian Y and Yang R. A novel and selective inhibitor of PKC zeta potently inhibits human breast cancer metastasis in vitro and in mice. Tumour biology : the journal of the International Society for Oncodevelopmental Biology and Medicine. 2016; 37(6):8391-8401.

8. Paul A, Danley M, Saha B, Tawfik O and Paul S. PKCzeta Promotes Breast Cancer Invasion by Regulating Expression of E-cadherin and Zonula Occludens-1 (ZO-1) via NFkappaB-p65. Sci Rep. 2015; 5:12520.

9. Tardaguila M, Mira E, Garcia-Cabezas MA, Feijoo AM, Quintela-Fandino M, Azcoitia I, Lira SA and Manes S. CX3CL1 promotes breast cancer via transactivation of the EGF pathway. Cancer Res. 2013; 73(14):4461-4473.

10. Park MH, Lee JS and Yoon JH. High expression of CX3CL1 by tumor cells correlates with a good prognosis and increased tumor-infiltrating CD8+ T cells, natural killer cells, and dendritic cells in breast carcinoma. J Surg Oncol. 2012; 106(4):386-392.

11. Tsang JY, Ni YB, Chan SK, Shao MM, Kwok YK, Chan KW, Tan PH and Tse GM. CX3CL1 expression is associated with poor outcome in breast cancer patients. Breast Cancer Res Treat. 2013; 140(3):495-504.

12. Husa AM, Magic Z, Larsson M, Fornander T and Perez-Tenorio G. EPH/ephrin profile and EPHB2 expression predicts patient survival in breast cancer. Oncotarget. 2016; 7(16):21362-21380.

13. Brantley-Sieders DM, Jiang A, Sarma K, Badu-Nkansah A, Walter DL, Shyr Y and Chen J. Eph/ephrin profiling in human breast cancer reveals significant associations between expression level and clinical outcome. PLoS One. 2011; 6(9):e24426.

14. Fox BP and Kandpal RP. A paradigm shift in EPH receptor interaction: biological relevance of EPHB6 interaction with EPHA2 and EPHB2 in breast carcinoma cell lines. Cancer Genomics Proteomics. 2011; 8(4):185-193.

15. Li X, Liu J, Wang Y, Zhang L, Ning L and Feng Y. Parallel underexpression of kallikrein 5 and kallikrein 7 mRNA in breast malignancies. Cancer science. 2009; 100(4):601-607.

16. Sidiropoulos KG, White NM, Bui A, Ding Q, Boulos P, Pampalakis G, Khella H, Samuel JN, Sotiropoulou G and Yousef GM. Kallikrein-related peptidase 5 induces miRNA-mediated anti-oncogenic pathways in breast cancer. Oncoscience. 2014; 1(11):709-724.

17. Pampalakis G, Obasuyi O, Papadodima O, Chatziioannou A, Zoumpourlis V and Sotiropoulou G. The KLK5 protease suppresses breast cancer by repressing the mevalonate pathway. Oncotarget. 2014; 5(9):2390-2403.

18. Orzol P, Nekulova M, Holcakova J, Muller P, Votesek B and Coates PJ. DeltaNp63 regulates cell proliferation, differentiation, adhesion, and migration in the BL2 subtype of basal-like breast cancer. Tumour biology : the journal of the International Society for Oncodevelopmental Biology and Medicine. 2016; 37(8):10133-10140.

19. Dang TT, Esparza MA, Maine EA, Westcott JM and Pearson GW. DeltaNp63alpha Promotes Breast Cancer Cell Motility through the Selective Activation of Components of the Epithelial-to-Mesenchymal Transition Program. Cancer Res. 2015; 75(18):3925-3935.

20. Ai L, Kim WJ, Alpay M, Tang M, Pardo CE, Hatakeyama S, May WS, Kladde MP, Heldermon CD, Siegel EM and Brown KD. TRIM29 suppresses TWIST1 and invasive breast cancer behavior. Cancer Res. 2014; 74(17):4875-4887.

21. Dukel M, Streitfeld WS, Tang TC, Backman LR, Ai L, May WS and Brown KD. The Breast Cancer Tumor Suppressor TRIM29 is Expressed via ATM-Dependent Signaling in Response to Hypoxia. J Biol Chem. 2016.
